# Simple, Sensitive and Simultaneous Determination of Free D3 and K2 Vitamins in Fortified Chicken Meat Products by LC-MS/MS with Electrospray Ionisation

**DOI:** 10.3390/foods15030570

**Published:** 2026-02-05

**Authors:** Mitja Križman

**Affiliations:** Laboratory for Food Chemistry, Department of Analytical Chemistry, National Institute of Chemistry, Hajdrihova 19, 1000 Ljubljana, Slovenia; mitja.krizman@ki.si

**Keywords:** lipophilic vitamins, vitamin D, vitamin K, meat products, fatty matrix, LC–MS, electrospray

## Abstract

A rapid and simplified LC–MS method was developed for quantifying vitamins D3 (cholecalciferol) and K2 (menaquinone-4 and menaquinone-7) in high-fat chicken meat products. Sample preparation involves a two-step solvent extraction followed by centrifugation. Efficient separation was achieved on a Gemini C18 column, and electrospray in positive mode was used for detection. Method validation confirmed good performance and reproducibility. The method was successfully applied to both fortified and unfortified chicken pâté samples. Owing to its simplicity, robustness, and sensitivity, this approach provides a practical and reliable means for quantifying fat-soluble vitamins in complex animal-derived matrices and can serve as a foundation for broader applications in high-fat food products.

## 1. Introduction

Vitamins from groups D and K are essential fat-soluble micronutrients involved in calcium metabolism and have complementary metabolic roles [1,2,3]. The crucial role of vitamins, especially vitamin D3, has been recognised for a long time [4]. Meat is an important dietary source of these vitamins. Due to their nutritional importance, many contemporary food products are also fortified with these vitamins [5,6,7]. Analytical procedures for determining these vitamins, especially vitamin D3, have long been established, beginning with thin-layer chromatography [8] or gas chromatography [9]. However, the vast majority of analyses to date have been based on high-performance liquid chromatography (HPLC). Earlier HPLC procedures used UV detection and usually required more involved sample preparation, such as liquid–liquid extraction, solid-phase extraction, and sample concentration, due to the low inherent concentrations of these vitamins in studied foods [10,11,12,13,14]. Modern analytical procedures for assaying these vitamins are still based on liquid-phase separations, but mass spectrometric (MS) detection is usually employed [15,16,17,18,19]. While the selectivity and sensitivity of MS detection allow for better instrumental performance, sample preparation is in many instances still laborious, mainly due to the need to remove potentially interfering fatty matrix in meat samples [20,21,22]. When hydroxylated metabolites of vitamin D (e.g., 25-hydroxycholecalciferol) are also involved in the analysis of biological tissues (such as meat), sample saponification is also required, involving even more elaborate sample preparation procedures [23,24], since hydroxylated forms of vitamin D3 have a much higher binding affinity to specific proteins [25]. In the case of milk and dairy samples, analyte derivatisation has also been employed [21,26]. In this context, relatively simple sample preparation procedures are only known when such vitamins are analysed from biological fluids like blood plasma, where sample preparation is generally limited to protein precipitation and solvent reconstitution [27,28,29,30], although in some cases derivatisation has also been used [29,31].

Vitamin K2, on the other hand, is involved in the so-called “vitamin K cycle”, in which a circular redox reaction occurs, and the vast majority of K2 is present in its oxidised (i.e., quinone) form [32]. Due to its high lipophilicity, it associates with various lipoproteins through non-covalent interactions [33]. Therefore, a simple extraction step (with subsequent solvent evaporation and redissolution, if needed) is sufficient for vitamin K [34], or a series of solvent evaporation and re-extraction steps [35]. In a highly lipophilic matrix, QuEChERS cleanup has also been used [36]. Beyond the domain of biochemical or comprehensive food studies, the need for a quick, robust and credible assessment of these vitamins is relevant to many food production facilities, particularly those producing meat and meat-derived products. Food products currently on the market are often fortified with these vitamins due to their recognised health effects [5,37,38,39].

In this study, an analytical procedure for the simple and rapid determination of fortified vitamin D3 (cholecalciferol), naturally occurring vitamin K2 (as menaquinone-4 or MK4), and fortified K2 (as menaquinone-7 or MK7) is presented. The aims of this analytical study were: (a) to simplify sample preparation, preferably to a single step; (b) to develop a reasonably short chromatographic separation suitable for sample solutions with a high-fat matrix; and (c) to deploy appropriate MS detection capable of handling relatively low analyte concentrations and a complex sample matrix.

The objective was to obtain a reliable, robust analytical tool for use in a production environment where input and output process controls must be performed rapidly, from raw input materials to the finished product, chicken pâté. Additionally, such an analytical procedure is required as a tool for monitoring product stability and homogeneity. This is particularly important when vitamins are fortified in the end product.

## 2. Materials and Methods

### 2.1. Samples

Samples of chicken pâté products were provided by a local food company participating in the project. Some of these pâté samples were fortified with vitamins D3 and K2 (in the MK-7 form).

### 2.2. Chemicals and Solutions

Demineralised water with a conductivity of 18 MΩ was obtained from a Milli-Q apparatus (Millipore, Billerica, MA, USA). All solvents, namely methanol and 2-propanol, were purchased from Merck (Darmstadt, Germany). The solvents used for sample extraction and solution preparation were of analytical (p.a.) grade, while those used for chromatography were LC–MS grade. Ammonium acetate (LC–MS grade) was purchased from Sigma-Aldrich (St. Louis, MA, USA). Cholecalciferol (vitamin D3) and menaquinone-4 (vitamin K2, MK-4) analytical standards were purchased from Sigma-Aldrich, and menaquinone-7 (vitamin K2, MK-7) was purchased as a USP reference standard. The working standard solution consisted of vitamins D3, K2–MK4, and K2–MK7, each at 15 ng/mL, prepared in 2-propanol/methanol (1:1, *v*/*v*).

### 2.3. Sample Preparation Procedure

Meat pâté samples were homogenised by hand mixing. Approximately 1 g of sample was weighed into a 50 mL volumetric flask, and 25 mL of methanol were added. The resulting mixture was sonicated in an ultrasonic bath for 10 min at 50 °C. The flask contents were then cooled to room temperature. 2-Propanol was added up to the flask mark, and the flask was mixed by inversion. The flask was then sonicated again for about 10 min at 50 °C. After cooling to room temperature, an aliquot (about 1.5 mL) from the flask was centrifuged for about 10 min at 13,000× *g* in an Eppendorf tube. The supernatant was then transferred into an HPLC vial for analysis. Throughout sample preparation, exposure to light was avoided by using either brown glassware or protecting the samples with aluminium foil, and by working under subdued light.

### 2.4. LC–MS/MS Method

The analytical work was performed using an LC–MS system comprising a TSQ Quantum mass spectrometer coupled to an Accela 600 HPLC system, both from Thermo Scientific (San Jose, CA, USA), and the data acquisition software Xcalibur v. 2.1. The selected column was a Gemini C18 (octadecyl silica) with dimensions of 50 mm × 2 mm i.d., 3 μm particle size (Phenomenex, Torrance, CA, USA), with the temperature set at 30 °C. Sample vials were thermostatted at 15 °C. The autosampler flush solvent was pure methanol. The flow rate during analysis was constant at 0.3 mL/min, and the injection volume was 5 μL. The column elution gradient consisted of three solvents: A—water with 5 mM ammonium acetate; B—methanol with 5 mM ammonium acetate; C—2-propanol. The gradient conditions are shown in Table 1. The total analysis run time was 15 min.

Mass spectrometric detection was performed using a heated electrospray (ESI) ion source in positive ion mode, set at +6 kV and a vaporiser temperature of 385 °C. The sheath gas, ion sweep gas, and auxiliary gas pressures were set at 10 psi, 0.3 psi, and 5 psi, respectively. The transfer capillary was set at 280 °C and the skimmer voltage at 5 V. The total MS acquisition time was 8 min. Analyte-specific parameters are shown in Table 2.

### 2.5. Method Validation

The developed LC–MS method was evaluated for several validation parameters to ensure successful quantification of analytes, including precision, accuracy, sensitivity, linearity, and stability. Injection precision was determined by four injections of the working standard solution. Extraction efficiency was assessed by three consecutive extractions of selected samples, and analyte recovery was compared with the combined recovery from all extraction steps. Accuracy was determined in four replicates by spiking homogeneous samples with standards at 50% concentration relative to the working standard solution, followed by the described sample preparation procedure. Repeatability and intermediate precision were also tested on a homogeneous sample. Four replicates were assayed for repeatability, while three replicates were assayed on each of three consecutive days for intermediate precision. Limits of detection (LOD) and quantitation (LOQ) were based on signal-to-noise ratios of 3 and 10, respectively, obtained from standard solutions. Linearity was checked in two replicates over the range of 10 to 200% concentration relative to the working standard solution, at a minimum of six points. Correlation coefficients were calculated with intercept values set to zero. Stability tests were conducted using a spiked sample solution kept at 15 °C in the dark for approximately 24 h (autosampler).

## 3. Results and Discussion

### 3.1. Sample Preparation Strategy

From the outset of method development, the aim was to use the simplest possible sample preparation approach, thereby avoiding unnecessary and laborious steps. Procedures such as solvent evaporation, sample reconstitution, re-extraction, and solid-phase extraction were intentionally omitted. A sample weight to solvent volume ratio of 1:50 was found to be optimal, providing good extraction efficiency (recovery) while maintaining adequate detection sensitivity in the working sample solutions. The stepwise addition of solvents during extraction (first methanol, then 2-propanol) was also found to be effective, as methanol provided sufficient extraction strength for the analytes (at this sample weight to solvent volume ratio) while preventing complete dissolution of fatty substances in the sample solution. Interestingly, the simultaneous use of methanol and 2-propanol during extraction proved less effective, resulting in prolonged extraction times and higher levels of chemical background noise in the chromatograms. This may be attributed to the concurrent dissolution of fatty substances, which partially hinders the dissolution of analytes. Mild heating during extraction further improved extraction efficiency, with 50 °C providing the optimum balance between extraction efficiency, short extraction times, and low chemical noise. The addition of 2-propanol in the second extraction step was primarily a preventive measure to stabilise the sample solutions for further analysis, preventing the precipitation of solutes of interest after a certain period (i.e., improving solution stability after about 24 h or more). A feasible explanation for this is the relatively low solubilising power of methanol alone as a solvent for lipophilic compounds. While this may not be an issue for chicken meat, which typically has a fat content below 15%, in the case of chicken pâté, with a fat content around 30%, some matrix-related issues, such as fat precipitation, have arisen without the addition of 2-propanol. Centrifugation of the sample solution was necessary for debris removal and also proved to be more practical and economical than filtration.

### 3.2. Analyte Selection and Chromatographic Conditions

During the method development phase, several HPLC stationary phases were evaluated for selectivity, focusing primarily on columns with low to mid-range carbon loads, while avoiding high carbon load columns due to potential issues with elution and excessive retention of highly lipophilic compounds (e.g., lipids). Columns with lower carbon loads were inadequate for efficient analyte separation, whereas columns with higher carbon loads presented problems with matrix elution consistency and resulted in longer run times. The Gemini C18 stationary phase, with a carbon load of approximately 14%, was ultimately selected based on its good selectivity and the consistent results obtained after many repeated injections, indicating complete and consistent elution of potentially interfering matrix compounds. The final elution phase before column re-equilibration, consisting mainly of 2-propanol (85% C), is crucial for fatty matrix removal from the column and for achieving consistent results. Although the fatty sample matrix is an important consideration in this case, and HILIC separation mode could theoretically be a more appropriate approach for such a set of analytes, the choice of reversed-phase HPLC separation was made on a practical basis, due to potentially better compatibility of sample extraction solvents with a reversed-phase separation, rather than with a HILIC one, as alcohols are used for extraction.

**Figure 1:**
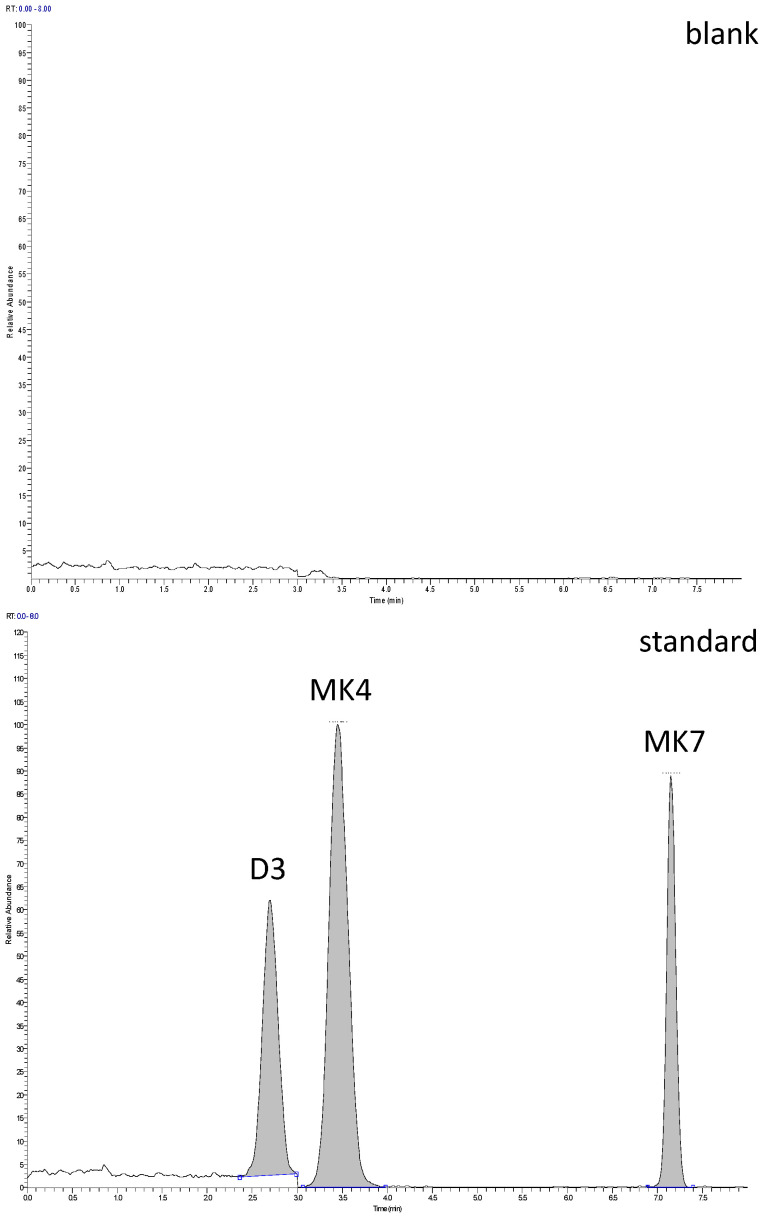

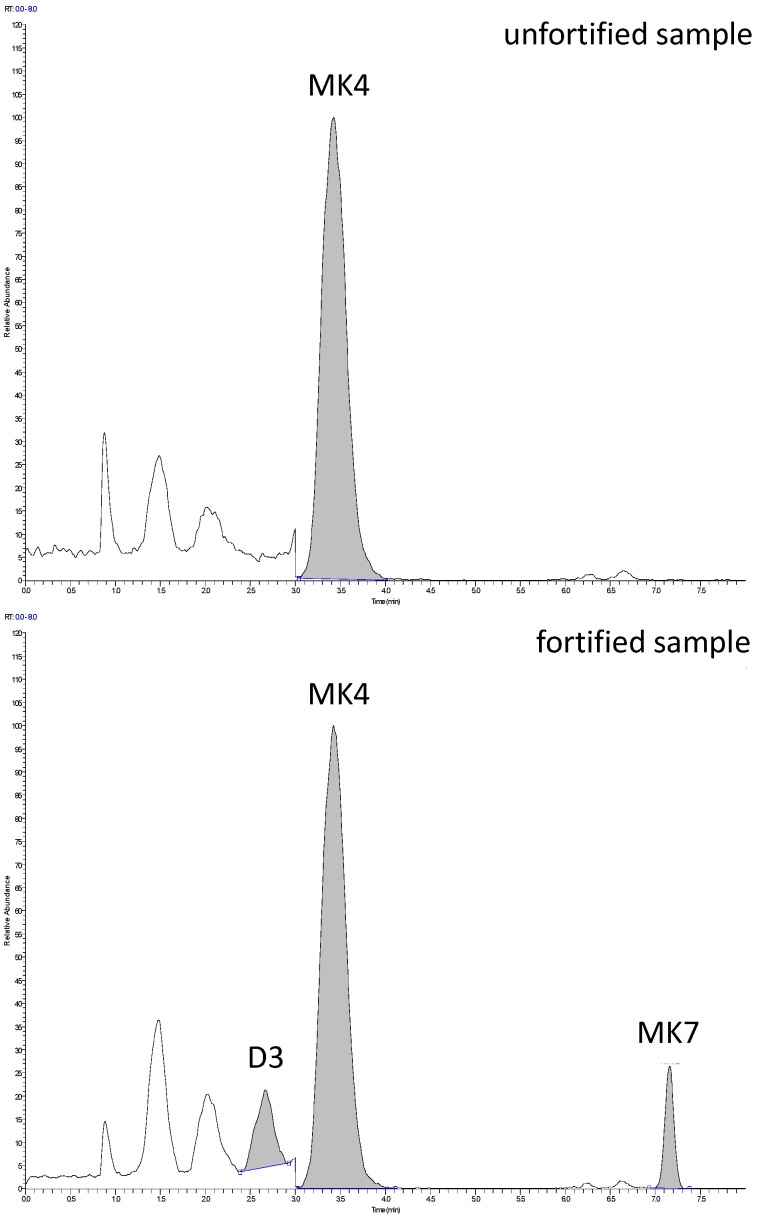
Chromatograms of blank, standard solution, unfortified pâté sample and pâté fortified with D3 and K2–MK7 (**top** to **bottom**).

For practical reasons, only the native form of vitamin D3 (cholecalciferol) was analysed, excluding its metabolites (with 25OH–D3 having the highest concentration among them). In addition to absolute concentrations, previous studies indicate significant differences in the ratio between vitamin D3 and 25OH–D3 among different types of meat [20,34], while this ratio is quite predictable within a specific type of meat. Studies on chicken meat samples [20,40,41,42] indicate that the majority of vitamin D3 is present as cholecalciferol, accounting for approximately 50–60% of total vitamin D3 in unfortified meat. Additionally, the vast majority of vitamin D3 in the studied samples is present in its native and free form due to fortification. It must also be mentioned that, in general, the absolute content of unfortified vitamin D3 can be highly variable. Values as high as 4.5 µg/100 g of free vitamin D3 in chicken meat have also been reported [40]. Examples of chromatograms are shown in Figure 1.

### 3.3. MS Detection

Vitamins D3 and K2 both lack ionisable groups. Early LC–MS methods used thermospray (TS) ionisation [19], particle beam (PB) ionisation [15,16], and later atmospheric-pressure chemical ionisation (APCI) [17,18,20,22,27]. Only more recently, and with fewer reports to date, has electrospray (ESI) ionisation also become a valid option in this context [21,28,29]. With technical developments and improvements, ESI provides a viable option for ionisation of non-polar compounds as well, provided they are detected as adducts with suitable ions [43,44], while also offering lower levels of chemical noise. With ESI, in some cases, extreme measures have been taken to obtain well-defined adducts, for example, by using methylamine in the mobile phase [21]. In other cases, a range of adducts has been observed, from protonated species to ammoniated and sodiated adducts, most likely originating from matrix interferences [45].

In this study, to keep the analytical procedure simple and minimise sources of error, only ammonium acetate was used as a mobile-phase modifier for ionisation. The most prevalent ion species observed were protonated target analytes, with no appreciable detection of ammoniated adducts (as theoretically expected), even during method development. A plausible explanation for the almost exclusive presence of protonated species is the use of a relatively high vaporisation temperature (385 °C) and electrospray voltage (6 kV) in the ion source, which probably favours the stability of protonated rather than ammoniated species. At lower vaporisation temperatures, ammoniated species were also present at low levels, but the background signal (chemical noise) was significantly higher. At higher vaporisation temperatures, the analyte (parent ion) signals began to decrease, indicating that the selected vaporisation temperature is optimal, at least for this specific case. Interestingly, with the use of formic acid as a mobile-phase modifier (instead of ammonium acetate), the detector responses for analytes were slightly lower. This may be attributed to several factors: on one hand, the higher conductivity of such a mobile phase, and on the other, analyte-modifier and/or matrix-modifier interactions, which would require a more detailed mechanistic study beyond the scope of this work. Regardless of the ESI conditions, the presence of analyte adducts with sodium and potassium was also expected due to the complexity of the matrix. Contrary to predictions, no such adducts were observed. This implies separation efficiency not only in terms of analyte selectivity, but also in terms of matrix interference removal.

**Figure 2:**
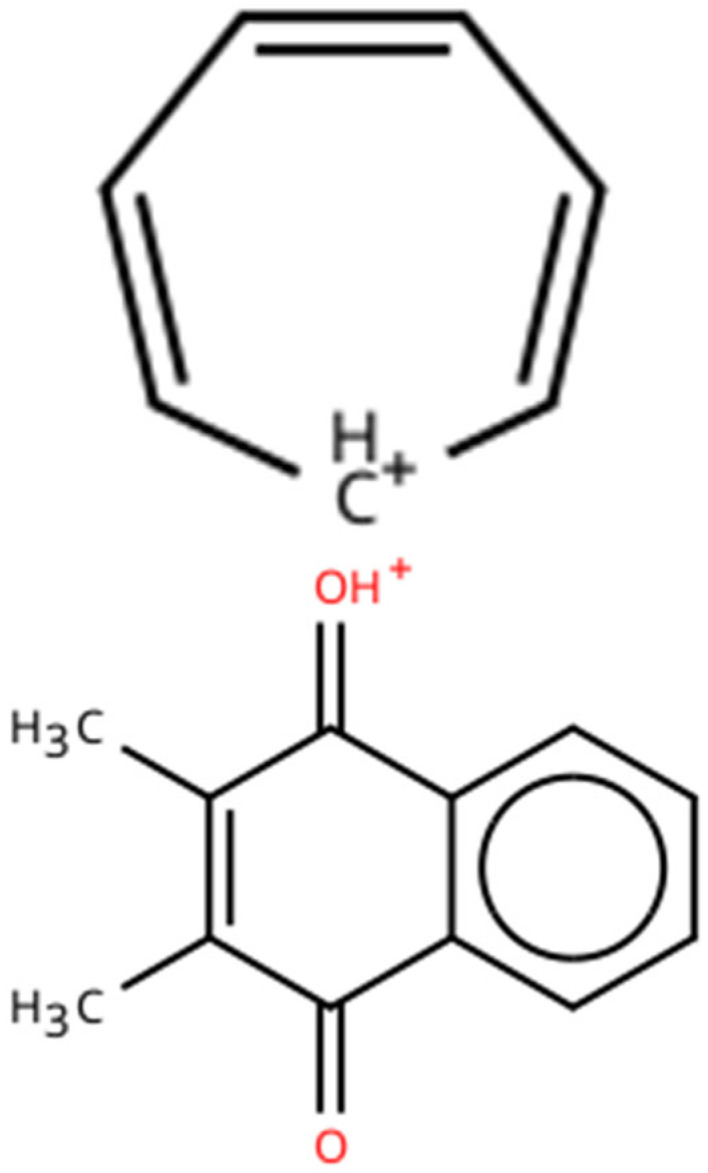
Proposed structures of observed product ions. **Top**: tropylium cation (*m*/*z* 91). **Bottom**: protonated methyl menadione (*m*/*z* 187).

In contrast to most related studies, the fragmentation conditions in this study were set relatively high in terms of collision energy, resulting primarily in smaller fragment (product) ions. This approach was chosen deliberately during method development, as the large difference in *m*/*z* values between precursor and product ions appeared to lower signal background noise, thereby improving sensitivity. The main product ion observed for D3 was at *m*/*z* 91, while for both K2–MK4 and K2–MK7 the product ion was at *m*/*z* 187. Product ion spectra for all three analytes under experimental conditions are shown in Figures S1–S3. The product ion at *m*/*z* 91 most likely corresponds to the tropylium cation, which can be formed by molecular rearrangement, also from non-aromatic moieties [46,47,48,49,50]. While tropylium ions as a fragment species were originally found in electron ionisation spectra, their occurrence has also been confirmed in collision-induced dissociation spectra from parent ions originating from positive electrospray ionisation [51,52]. The product ion at *m*/*z* 187 is most likely the protonated species of methyl menadione [53,54]. The proposed ionic species are shown in Figure 2. To simplify the procedure and reduce operating costs, no isotope-labelled standards were used, as the validation data showed good figures of merit (Section 3.4). For MS detection, only single ion transitions were monitored (single reaction monitoring, SRM), recording only quantifier product ions and not the so-called qualifier ions. This decision was made to maximise MS detection sensitivity, partly because the instrument used (TSQ Quantum Ultra) tends to drastically reduce sensitivity when multiple transitions are recorded simultaneously. Nevertheless, the method offers very good sensitivity at the same or a higher level compared to similar methods (Table A1).

### 3.4. Method Validation, Performance and Applicability

The validation data indicate good figures of merit for the proposed methodology, with no issues regarding any performance parameters, even though quantification was based solely on external standards. The use of isotope-labelled internal standards was beyond the scope of the method, as good validation data were already obtained as described, without unnecessarily increasing the method’s operating costs. The method has been tested on chicken meat-derived pâté products, which have very complex matrices containing additional animal fat, vegetable oils, and other components. Therefore, it is assumed, although not tested (due to project constraints), that this methodology could also serve as a good starting point for other animal-derived raw materials and products high in fatty matrix and with similar vitamin levels. The validation results are shown in Table 3.

### 3.5. Sample Analyses

Examples of analytical results from different pâté samples are shown in Table 4. Although all results fall within the expected or specified ranges, they demonstrate variability in measured vitamin levels, despite the thorough industrial processes used in manufacturing. This variability can be attributed both to batch-to-batch variation in raw materials and to inhomogeneity issues arising during vitamin fortification in the mixing phase of production. The quantities of vitamins required for fortification are quite small, and although they are pre-solubilised in edible oil, their distribution within a production batch must be carefully monitored through analysis. In addition to monitoring vitamin stability, homogeneity monitoring was also a key task in the development of the method.

### 3.6. Potential Limitations and Future Prospects

The method relies on a simple, yet robust, sample extraction procedure, which inevitably co-extracts a large portion of the fatty matrix. Therefore, as a trade-off, much of the analytical run time is devoted to matrix elution and column conditioning, prolonging the analysis as the main drawback. Although the developed method was primarily intended as a quality control tool within the production environment, its relative simplicity means it could be adapted—particularly in terms of sample preparation (i.e., solvents and extraction conditions)—for other types of animal-derived raw materials and final products, such as dairy or other types of meat.

## 4. Conclusions

The presented LC–MS method offers a simple and efficient approach for the analysis of vitamins D3 and K2 in chicken meat products with matrices containing high fat content (around 30%). Although the method has not yet been applied to plain meat samples (as this was beyond the scope of the project), it is reasonable to assume that it can also be used to analyse native MK4 content in meat. Furthermore, in cases of high vitamin D3 levels (i.e., above 0.6 µg/100 g), it can also be used for such analyses. Its main advantages are simplicity, sensitivity, and robustness.

## Figures and Tables

**Table 1:** Mobile-phase gradient program.

Time (min)	% A	% B	% C
0.0	5	95	0
4.0	5	95	0
4.5	5	10	85
10.0	5	10	85
10.1	5	95	0
15.0	5	95	0

**Table 2:** Analyte-specific MS parameters.

Time (min)	Parent *m*/*z*	Product *m*/*z*	Collision Energy (V)	Tube Lens (V)	Collision Gas Pressure (mTorr)	Analyte
0–2.67	385.1	91.1	50	91	1.7	D3
2.67–4.5	445.2	187.0	22	82	1.7	MK4
4.5–8.0	649.5	187.0	32	115	1.7	MK7

**Table 3:** Method validation parameters.

Analyte	Injection Precision (% RSD, n = 4)	Accuracy (%, n = 4)	Extraction Efficiency (%) *	Repeatability (% RSD, n = 4)	Intermediate Precision (% RSD, n = 9)	LOD ** (ng/g)	LOQ ** (ng/g)	Regression Coefficient (r)	Stability 24 h (%)
D3 K2–MK4 K2–MK7	1.38	86.6 ± 5.1	-	5.87	6.48	2.4	6.1	0.9981	98.0
	3.81	104.9 ± 4.8	93.8	5.17	5.71	5.9	19.8	0.9991	94.3
	2.04	88.7 ± 3.6	94.6	2.94	7.55	4.2	15.7	0.9983	93.9

* D3 has not been assessed for extraction efficiency, since the second consecutive extraction of samples gave no detectable peaks in unspiked samples. ** determination of LOD and LOQ was based on extrapolation of signal-to-noise responses, expressed as ng in g of sample; LOD—limit of detection; LOQ—limit of quantitation.

**Table 4:** Sample analyses. The samples have been analysed in duplicates. The values are expressed in µg per 100 g of sample.

Analyte	D3	K2–MK4	K2–MK7
Pâté Type	(µg/100 g)	(µg/100 g)	(µg/100 g)
Unfortified	n.d	48.3 ±1.6	n.d
Unfortified	2.1 ± 0.1	63.8 ± 3.7	2.6 ± 0.3
Unfortified	0.9 ± 0.1	53.1 ± 2.9	1.8 ± 0.2
Unfortified	3.4 ± 0.2	61.9 ± 2.4	2.4 ± 0.1
Fortified	13.8 ± 0.6	58.9 ± 3.6	18.2 ± 0.4
Fortified	8.1 ± 0.4	42.7 ± 2.3	13.6 ± 0.7
Fortified	22.1 ± 0.7	74.6 ± 1.7	28.0 ± 0.7
Fortified	8.2 ± 0.3	90.4 ± 2.4	12.2 ± 0.8

n.d.—not detected.

## Data Availability

The original contributions presented in the study are included in the article/Supplementary Materials. Further inquiries can be directed to the corresponding author.

## Supplementary Materials

The following supporting information can be downloaded at: https://www.mdpi.com/article/10.3390/foods15030570/s1, Figure S1: Fragmentation spectra of cholecalciferol (vitamin D3), Figure S2: menaquinone-4, Figure S3: menaquinone-7.

## Appendix A

In Table A1, a comparison between LC–MS methods referenced for analysis of vitamins D and K has been carried out regarding sample type(s), sample preparation procedure, analytical run time(s), and limits of detection (LOD) and quantification (LOQ).

**Table A1:** Comparison among different published LC–MS methods. * refers to the analyte amount injected on column (recalculated from published data, as needed); n.a.—not available; Apx.—approximately; SPE—solid phase extraction; SLE—supportive liquid extraction; LLE—liquid–liquid extraction.

Analytes	Sample Type(s)	Sample Preparation Procedure	LC(–MS) Run Time (min)	LOD (pg) *	LOQ (pg) *	Reference
Vit. A, D3, E	dietetic infant formula, infant cereals	Saponification, SPE, solvent reconstitution	30	80 for D3	200 for D3	[17]
Vit. K1, K2-MK4, K2-MK7	Human plasma	Solvent extraction, SPE, solvent reconstitution	75	n.a.	1.2 for K1 1.5 for MK4 2.4 for MK7	[18]
Vit. D3, 25OH-D3	Fish, meat, eggs	Saponification, extraction, SPE, solvent reconstitution, preparative chromatography	20	508 for D3	Apx. 2000 for D3	[20]
Vit. D3, 25OH-D3	Meat, eggs, dairy prod.	Saponification, extraction, SPE, derivatisation	18	n.a.	n.a.	[21]
Vit. D2, 25OH-D2, D3, 25OH-D3	Various meats	Saponification, extraction, SPE, solvent reconstitution	35	Apx. 280 for D3	Apx. 470 for D3	[22]
Vit. D2, 25OH-D2, D3, 25OH-D3	Human and cow milk	Solvent extraction, SPE, solvent reconstitution, derivatisation	17	n.a.	1.9 for D3 7.7 for 25OH-D3	[26]
Vit. 25OH-D2, 25OH-D3	Human serum/plasma	Solvent extraction/precipitation, solvent reconstitution	10	Apx. 45 for 25OH-D2 Apx. 90 for 25OH-D3	Apx. 225 for 25OH-D2 Apx. 450 for 25OH-D3	[27]
Vit. 25OH-D2, 25OH-D3 and others	Human serum	Solvent extraction/precipitation, SLE, solvent reconstitution	8	n.a.	Apx. 2.2 for 25OH-D2 Apx. 4.4 for 25OH-D3	[28]
Vit. 25OH-D2, 25OH-D3 and others	Human serum	Solvent extraction/precipitation, solvent reconstitution, derivatization, solvent reconstitution	5.5	0.04 for 25OH-D2 0.2 for 25OH-D3	0.2 for 25OH-D2 1 for 25OH-D3	[29]
Vit. D2, 25OH-D2, D3, 25OH-D3 and others	Human plasma	Solvent extraction/precipitation, solvent reconstitution, LLE, solvent reconstitution	8	1.3 for D2 1 for D3 5.3 for 25OH-D3	4.5 for D2 3.2 for D3 17.6 for 25OH-D3	[30]
Vit. D3, 25OH-D3 and others	Human serum	Solvent extraction/precipitation, solvent reconstitution, LLE, derivatization, solvent reconstitution	14.5	n.a.	8 for D3	[31]
Vit. K1, K2-MK4, K2-MK7	Various foods	Solvent extraction, centrifugation, solvent reconstitution	25	5.1 for MK4 10.0 for MK7	17.6 for MK4 33.3 for MK7	[34]
Vit. K1, K2-MK4, K2-MK7, K2-MK9	Human and rat serum	Solvent extraction/precipitation, miniaturised LLE, solvent reconstitution	8.5	0.8 for MK4 0.5 for MK7	1.3 for MK4 0.8 for MK7	[35]
Vit. D2, D3, K1, K2-MK4	Nanoemulsions, fortified yogurt	QuEChERS	6	3.8 for D2 0.9 for D3 0.5 for MK4	11.6 for D2 2.6 for D3 1.5 for MK4	[36]
Vit. D3, K2-MK4, K2-MK7	Chicken pâté	Solvent extraction, centrifugation	15	0.25 for D3 0.6 for MK4 0.4 for MK7	0.6 for D3 2 for MK4 1.6 for MK7	Present work
