# Supplementary material for: Simple, Sensitive and Simultaneous Determination of Free D3 and K2 Vitamins in Fortified Chicken Meat Products by LC-MS/MS with Electrospray Ionisation
Source: Foods. 2026 Feb 5;15(3):570. doi: 10.3390/foods15030570 (PMC12897166; doi:10.3390/foods15030570)
Supplement: Supplementary file 1 [file foods-15-00570-s001.zip › foods-4076966-supplementary.pdf]

Supplementary material

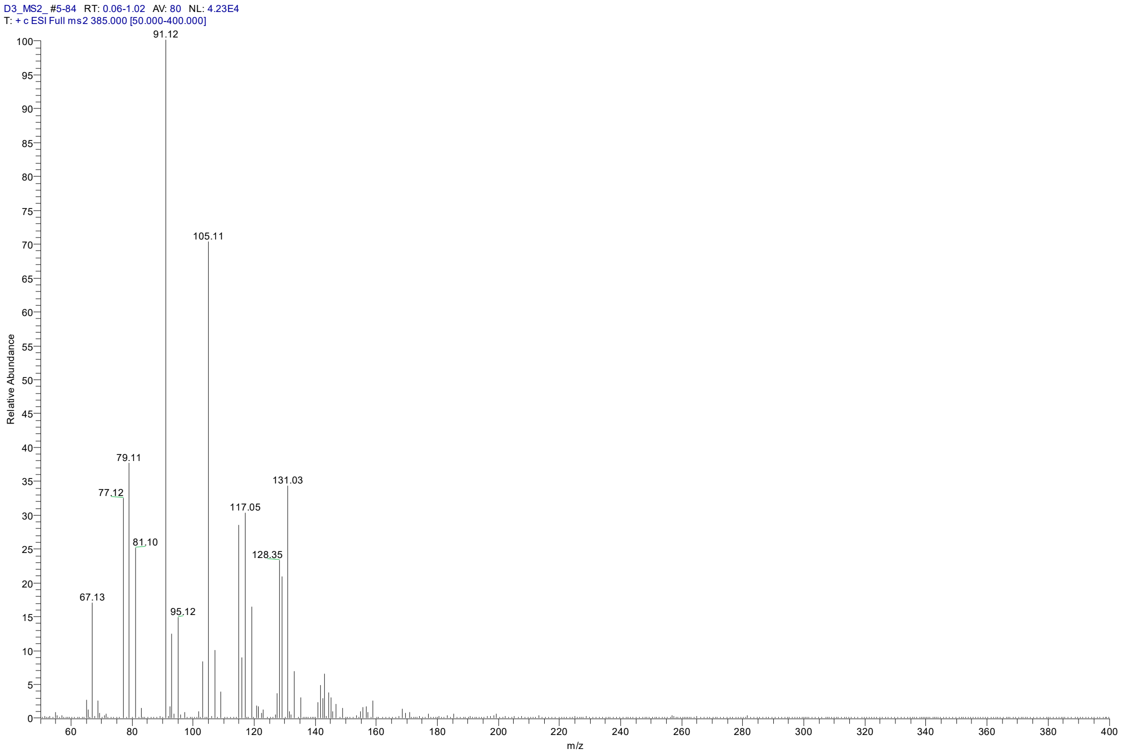

Figure S1. Collision-induced fragmentation spectrum of vitamin D3 (parent ion m/z 385.1) under experimental conditions: collision gas pressure 1.7 mTorr; collision energy 50 V

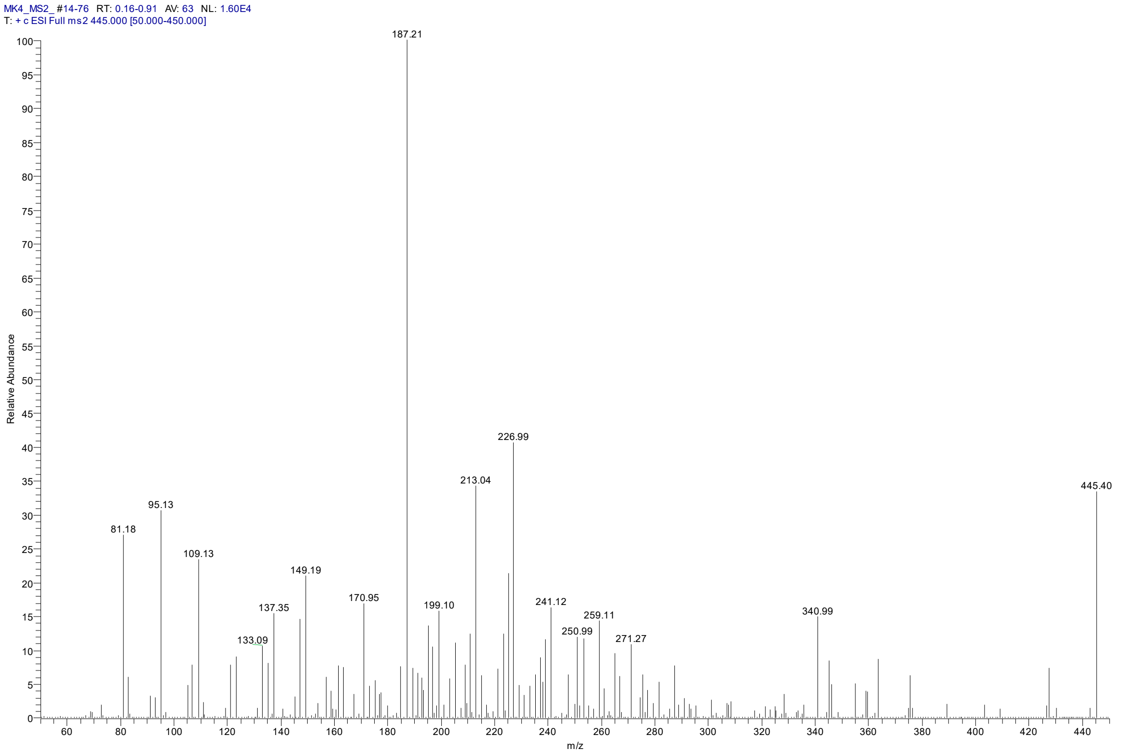

Figure S2. Collision-induced fragmentation spectrum of menaquinone-4 (parent ion m/z 445.2) under experimental conditions: collision gas pressure 1.7 mTorr; collision energy 22 V

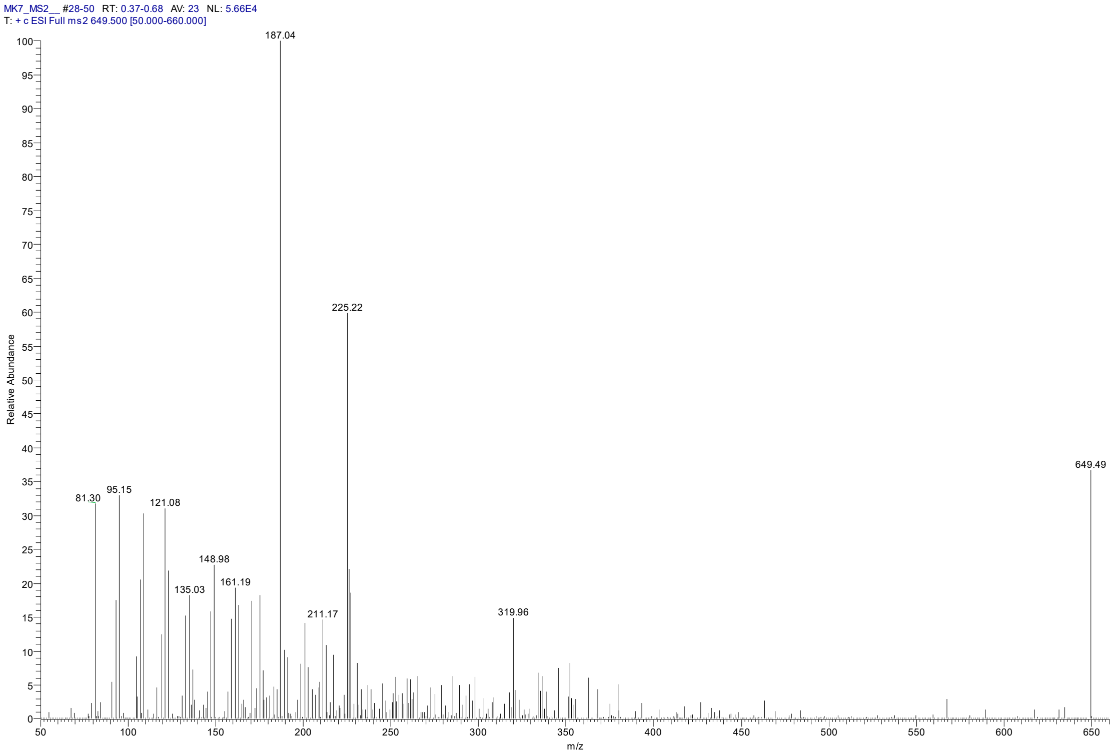

Figure S3. Collision-induced fragmentation spectrum of menaquinone-7 (parent ion m/z 649.5) under experimental conditions: collision gas pressure 1.7 mTorr; collision energy 32 V
